# Unlocking the monolithic integration scenario: optical coupling between GaSb diode lasers epitaxially grown on patterned Si substrates and passive SiN waveguides

**DOI:** 10.1038/s41377-023-01185-4

**Published:** 2023-06-16

**Authors:** Andres Remis, Laura Monge-Bartolome, Michele Paparella, Audrey Gilbert, Guilhem Boissier, Marco Grande, Alan Blake, Liam O’Faolain, Laurent Cerutti, Jean-Baptiste Rodriguez, Eric Tournié

**Affiliations:** 1 IES, University of Montpellier, CNRS, F-34000 Montpellier, France; 2 Department of Electrical and Information Engineering, Polytechnic University of Bari, 4 Via E. Orabona, IT- 70126 Bari, Italy; 3 Tyndall National Institute, Lee Maltings Complex, Dyke Parade, IR-T12R5CP Cork, Ireland; 4 Centre for Advanced Photonics and Process Analysis, Munster Technological University, Bishopstown, IR-T12P928 Cork, Ireland

**Keywords:** Semiconductor lasers, Silicon photonics

## Abstract

Silicon (Si) photonics has recently emerged as a key enabling technology in many application fields thanks to the mature Si process technology, the large silicon wafer size, and promising Si optical properties. The monolithic integration by direct epitaxy of III–V lasers and Si photonic devices on the same Si substrate has been considered for decades as the main obstacle to the realization of dense photonics chips. Despite considerable progress in the last decade, only discrete III–V lasers grown on bare Si wafers have been reported, whatever the wavelength and laser technology. Here we demonstrate the first semiconductor laser grown on a patterned Si photonics platform with light coupled into a waveguide. A mid-IR GaSb-based diode laser was directly grown on a pre-patterned Si photonics wafer equipped with SiN waveguides clad by SiO_2_. Growth and device fabrication challenges, arising from the template architecture, were overcome to demonstrate more than 10 mW outpower of emitted light in continuous wave operation at room temperature. In addition, around 10% of the light was coupled into the SiN waveguides, in good agreement with theoretical calculations for this butt-coupling configuration. This work lift an important building block and it paves the way for future low-cost, large-scale, fully integrated photonic chips.

## Abstract (web summary)

Artist view of a GaSb diode laser grown on a Si-photonics integrated circuit, with the emitted light coupled into passive SiN waveguides.

## Graphical abstract

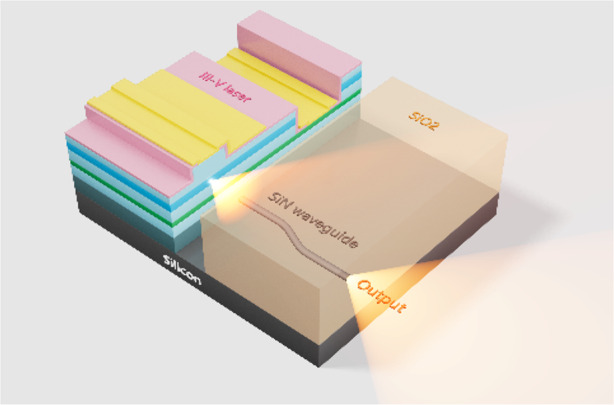

## Introduction

There is a soaring demand for Si photonics chips for applications such as data/telecom, optical interconnects, quantum technologies, or on-chip optical sensors, to name a few^1–8^. In this context, SiN-based waveguides have recently emerged as a favorable platform thanks to their superior high-power handling capability, wider transparency, larger cross-section, and lower loss as compared to Si^9–12^. In addition, various options are available to fabricate efficient electro-optic modulators in this technology through atomic layer deposition or transfer printing of nonlinear crystals^13–15^. Now, the indirect bandgaps of Si and Ge and the still-too-low performances of GeSn devices despite impressive progress^16–18^, make the integration of III–V semiconductor lasers with passive Si photonic integrated circuits (PICs) the next vital step toward fully integrated Si photonics chips^19–24^. The most mature integration strategy nowadays is heterogeneous integration, where III–V heterostructures are first grown on their native substrate before being bonded onto the Si PICs and processed into devices^25–31^. Commercial products have even entered the market (https://www.intel.com/content/www/us/en/architecture-and-technology/silicon-photonics/silicon-photonics-overview.html). Whatever the application, however, there is evidence that the direct epitaxy of the III–V semiconductor laser heterostructures on the Si PICs could surpass the heterogeneous strategy on a mid-to-long-term basis in terms of integration density and economic perspective^32,33^. In addition, the heterogeneous approaches require etching away the original III–V substrate, an unsustainable practice in the long term.

Given these perspectives, the direct growth of III–V lasers on Si substrates has been extensively studied in the last decade, and much progress has been made on the epitaxial integration of a variety of lasers emitting from the visible to the mid-infrared through the near-infrared^23,34–45^. Still, all epitaxial lasers on Si reported to date are discrete devices that have been grown on bare Si wafers and not on PICs. The next challenge is to combine epitaxial lasers and Si-photonics PICs, and to couple light from the active III–V structures to the passive Si-based devices. The growth of III–V semiconductors on Si inevitably results in different types of structural defects detrimental to device performances^46–51^. Although various strategies have been developed to avoid them or attenuate their impact, defect management requires growing relatively thick (1–5 µm) buffer layers underneath the laser structure. While this is no problem for discrete III–V-on-Si optoelectronic devices, these buffer layers prevent implementing evanescent light coupling into passive waveguides, as traditionally used in heterogeneous integration^10,25,26,29,52^. An alternative option would be to use butt coupling^53,54^ that has recently been explored to couple transfer-printed lasers^55,56^ or epitaxial III–V photodetectors^57^ with waveguides.

The epitaxial butt-coupling approach, however, poses challenges since the fabrication and processing conditions of the III–V and Si-based materials are only marginally compatible. As the lowest loss SiN waveguides require deposition and treatment at high temperatures (e.g., by Liquid Phase Chemical Vapor Deposition), there is no other choice than fabricating first the Si-photonics PICs, patterning the PIC wafers to define the epitaxy areas, and then epitaxially growing and processing the III–V laser structure. There is thus a need to develop methods to grow III–V materials on patterned wafers and to fabricate devices from the material grown in the recessed areas, without compromising the PIC quality. Note that solving this issue will also provide a way to fabricate butt-coupled epitaxially integrated photodetectors, for the laser devices can be used as photodetectors under reverse biasing.

**Fig. 1:**
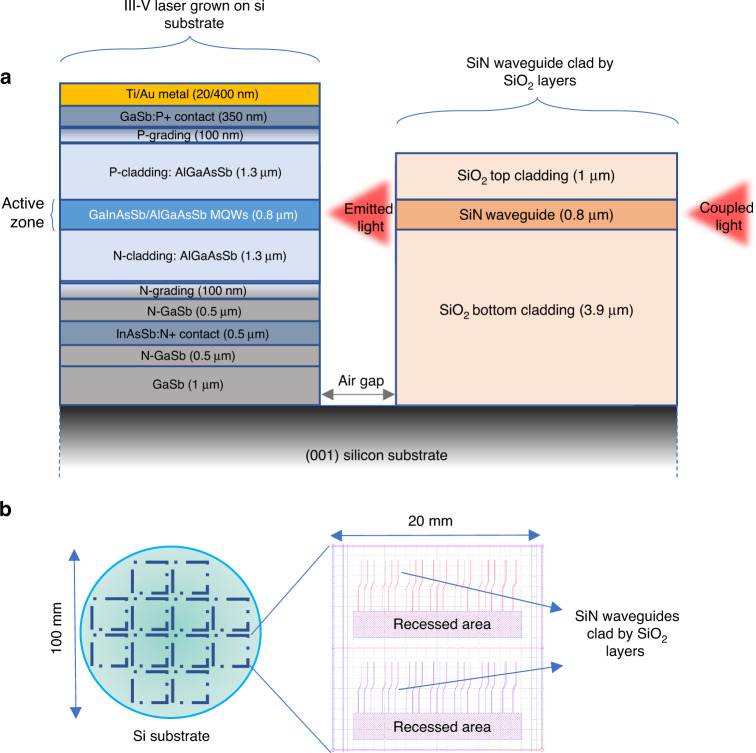
Overview of the integrated devices. **a** Cross-section sketch of the final device: the light emitted by the active zone of the epitaxially integrated III–V laser is coupled into the SiN waveguide. **b** Schematic of the Si-photonics PICs used in the experiments: 100-mm Si wafers are first processed to form the SiN waveguides clad by SiO_2_ layers. The pattern is organized in 20 × 20 mm^2^ square. The dielectric stack is then etched down to the Si substrate to open the recessed areas where the III–V lasers will be epitaxially grown. Then the wafers are diced to give 20 × 20 mm^2^ dices for epitaxy

In this work, we propose and explore strategies to overcome these challenges and we demonstrate a III–V laser grown on a patterned Si photonics platform with light coupling into passive SiN waveguides. Figure 1a presents the final device configuration with the respective materials. As a case study, we chose to integrate GaSb diode lasers (DLs) designed to emit near 2.3 µm, a wavelength of interest for trace gas sensing^58,59^ or LIDAR applications^60^. Figure 1b schematically represents the Si photonics platform used for these proof-of-concept experiments. Starting from 100-mm (001) Si wafers with a residual offcut of ∼0.5° in the [110] direction, 20 × 20 mm² dies were defined. Each die supported two series of S-shape SiN waveguides of various widths (10–20 µm) clad by SiO_2_ layers, and two recessed areas for laser growth. After fabrication, the original 100-mm wafers were diced, and each die could be used as an individual Si photonics platform.

## Results

### Design and fabrication of the Si photonics platform

The crucial point during Si PIC fabrication is to avoid damaging the Si crystal quality, for the GaSb epitaxy is extremely sensitive to the Si surface organization^61^. Thus, 3.9 µm of oxide was realized through thermal oxidation of silicon wafers, followed by the deposition of the Silicon Nitride (800 nm) and a 1000 nm top cladding silicon dioxide layer using PECVD. A photoresist was then spin-coated and the patterns were defined using photolithography. To avoid compromising the GaSb growth, it was again vital to ensure that the crystalline surface of the Si wafer was not damaged by the oxide etching. We thus employed a two-step process to create the recess in the thermal oxide: first, the pattern was etched ~95% of the way through the oxide/SiN stack using reactive ion etching, then the remaining oxide was removed using hydrofluoric acid wet etching. A one-step dry etch would have invariably damaged the Si surface. In contrast, hydrofluoric acid wet etching being isotropic, wet etching of the entire thickness of the stack would have resulted in a concave dielectric facet. Our approach provided a close-to-vertical dielectric facet (angle <10°) together with a near-perfect silicon substrate as a starting point for the growth. More details on the Si photonics platform fabrication can be found in “Materials and methods”.

### Epitaxy of the GaSb DL in the recessed area and material properties

As mentioned above, the epitaxy of III–V semiconductors on Si is plagued by defects, among which are the so-called antiphase boundaries (APBs) that act as shorts in devices. Although various strategies have been proposed to avoid these defects, including complex pre-processing of the Si wafers^24,62^, we have previously established a comparatively simple growth procedure to bury the APBs within the buffer layer^61^. This allowed us to demonstrate a variety of GaSb-based epitaxial MIR lasers grown by solid-source molecular-beam epitaxy (MBE) on plain Si substrates^23,41,42,44^. The key point is to obtain a perfectly organized starting Si surface. In this work, we applied a two-step growth procedure. First, a 1 µm thick, nominally undoped, GaSb buffer layer was grown by MBE on the Si PICs, based on the approach previously developed for growth on plain substrates^61^. The Si PICs were heated up to ∼1000 °C under vacuum in the MBE system to remove the Si native-oxide layer in the recessed areas that arises from the fabrication procedure. Then, their temperature was decreased down to the growth initiation temperature (∼400 °C) to epitaxially grow 50 nm GaSb, before the temperature was raised to 500 °C to grow the remaining 950 nm GaSb. The SiO_2_/SiN/SiO_2_ stacks and GaSb-on-Si PIC templates were thoroughly inspected at the various stages to fine-tune the growth process to ensure an APB-free GaSb surface while preserving the Si PIC integrity. These GaSb-on-Si PIC templates were then transferred to another MBE reactor to grow the whole laser structure^41^. The active zone was made of two GaInAsSb quantum wells (QWs) confined by AlGaAsSb barrier layers and was designed to emit near 2.3 µm.

**Fig. 2:**
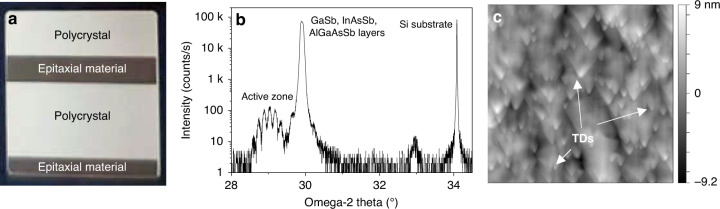
Structural properties of the epitaxial III–V on Si-PIC structures. **a** Picture of a 20 × 20 mm^2^ die after III–V epitaxy. The greyish areas correspond to the waveguide zones where polycrystal III–V material has been deposited on top of the dielectric material. The mirror-like areas are the recessed Si areas where single-crystal III–V material was grown. **b** Omega-2theta high-resolution diffraction scan of the laser structure, with the peaks identification, **c** 20 × 20 µm^2^ AFM image of the epitaxial laser surface. No threading APBs are seen. In contrast, threading dislocations, some of them being marked as TDs, are seen, which allows to estimate their density in the mid-10^7^ cm^−2^ range

The growth procedure for the DL part itself was similar to that used for GaSb DLs grown on their native substrate^63^. More details on the DL design and growth can be found in “Materials and methods”. Note that in the monolithic integration approach, the thickness of the buffer layer serves as a variable that can be adjusted at will to vertically align the DL active zone with the passive waveguide. A typical uncertainty of 3% on the growth rate results in a vertical misalignment lower than 100 nm, which, in turn, induces negligible insertion losses. However, MBE being a little selective, after epitaxy the dies exhibit alternating greyish and mirror-like areas (Fig. 2a) that correspond to polycrystal material deposited on the waveguide zones and to single-crystal material grown in the recessed area, respectively. The high-resolution X-ray diagram measured in the recessed area, however, shows well-defined diffraction features for all parts of the DL (Fig. 2b). In particular, the active zone pattern displays clear interference fringes arising from the periodicity of the multiple-quantum-well structure, which confirms that the active zone is well-defined with sharp interfaces. These results are similar to those achieved on DLs grown on plain Si wafers, which shows that the Si surface after PIC fabrication is comparable to that of a planar Si wafer. In addition, it demonstrates that the growth procedure is adapted to Si photonics platforms and that the material is of laser quality, despite the polycrystal at the recessed area/dielectric interface. Figure 2c shows a large view AFM image of the laser structure and confirms the absence of any APB reaching the sample surface. The roughness RMS is as low as 2.5 nm, whereas the threading dislocation density estimated from this image is in the mid-10^7^ cm^−2^.

### Laser processing

**Fig. 3:**
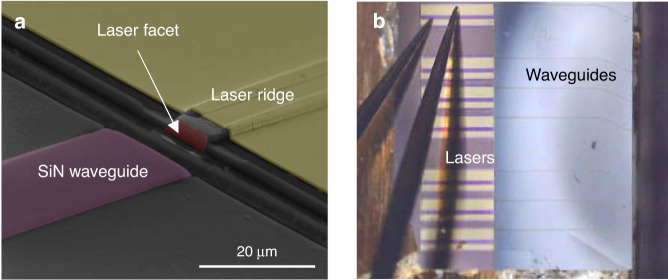
Pictures of processed devices. **a** SEM image showing a laser ridge with its etched facet and a passive waveguide. **b** Picture of a laser bar equipped with SiN waveguides ready to be tested on the probe station. The DLs are 1.5 mm long

As MBE is little selective, III–V materials deposit everywhere on the wafer, resulting in a thick III–V polycrystal layer deposited onto the amorphous SiO_2_/SiN/SiO_2_ layer stack and at the recessed-area/dielectric interface, as seen in Fig. 2a. The first step in the laser processing is therefore to remove the polycrystal without damaging either the laser or the waveguide structures. We developed a wet etch based on a C_6_H_8_O_7_:H_2_O_2_:HF:H_2_O solution that proved efficient to etch away the polycrystal. The epitaxial III–V single-crystal grown in the recessed areas must be protected to avoid any damage at this stage. We have used a photoresist as protective coating. The dies were then dipped into the etchant that removed all polycrystalline material, on top of the dielectric stack but also at the III–V/dielectric interface in the recessed area, without any apparent damage to both the III–V single-crystal epitaxial material and the dielectric stack. Pictures of similar interfaces taken right after epitaxy and after polycrystal removal are also shown in Fig. 2 of ref. ^53^. The polycrystal being removed, we processed the III–V heterostructure to fabricate etched-facet DLs using photolithography, based on the approach that we have previously established with GaSb DLs grown on plain Si wafers^64^. The similar performances of etched- and cleaved-facets DLs validate the etching process^64^. Still, here again the process flow had to be adapted to take into account the architecture of the PIC, in particular the fact that the DL material was grown in a recessed area. The whole process flow is described and documented with pictures in “Materials and methods”. Figure 3a shows a SEM image of a DL facet at the end of the process. It is smooth and vertical and we have previously demonstrated that such facets have a power reflectivity similar to cleaved facets^64^. Finally, the 20 × 20 mm^2^ dies were cleaved into two chips equipped with a laser bar and waveguides series (Fig. 3b). Each laser bar contains 8 DLs with 10 µm wide ridges and 1.5-mm long cavities. It is important to notice that the whole process (MBE growth + polycrystal removal + facet etching) results in the formation of an air gap between the laser and waveguide facets (Fig. 3a). In this sample, this air gap was about 15 µm wide. We will come back later to this aspect.

### Integrated diode laser properties

**Fig. 4:**
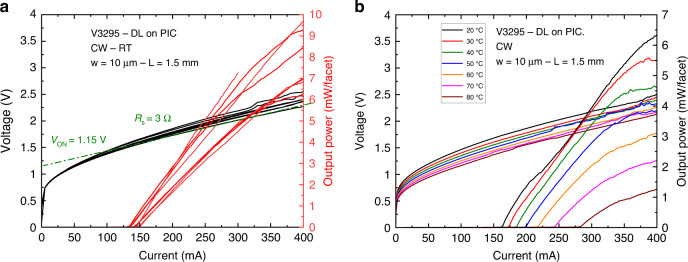
*L–I–V* characteristics of the DLs on a Si PIC. **a** *L–I–V* curves taken at room temperature in the CW regime for a series of 8 DLs. **b** *L–I–V* curves taken at different temperatures between 20 °C and 80 °C (setup limited) for a typical DL

The first step consisted in testing the DLs integrated on the PIC and benchmarking their performance against similar DLs grown on planar Si wafers. The light emission properties were measured on a probe station from the back etched facets in the CW regime (Fig. 3b) using a calibrated powermeter. Figure 4a shows the light–current–voltage (*L–I–V*) curves taken at room temperature from 8 DLs of a laser bar. All DLs did lase. The turn-on voltage is around 1 V, and the series resistance is near 3 Ω for all DLs. These values are comparable to that measured on GaSb-based DLs with a similar design, whether grown on GaSb^65–67^ or Si^23,41^ substrates. The threshold current intensity *I*_th_ varies between 135 and 150 mA, and the output power at 400 mA drive current lies between 7 and 10 mW, depending on the DL. This value spread is within the typical variations of our laser process. Figure 4b shows the evolution with the measurement temperature between 20 °C and 80 °C (limited by the experimental setup) of the *L–I–V* curves for a representative DL. Lasing is readily achieved in the whole temperature range with a threshold current intensity increasing from 163 to 283 mA. From this series of experiments, we deduced the corresponding *T*_0_ characteristic temperature, which represents the sensitivity of the laser threshold current to the operating temperature through ${I}_{{th}}(T)={I}_{0}{e}^{\frac{T}{{T}_{0}}}$, to be *T*_0_ = 110 K, a value again typical for GaSb DLs^67^.

Interestingly, the performance of the DLs epitaxially integrated on the PIC are similar to those grown on planar Si wafers^23,41^ which validates a number of important steps. First, the Si PIC fabrication is compatible with an epitaxy-compliant Si surface. Second, the MBE growth, polycrystal removal and laser fabrication are adapted to the particular sample architecture, in spite of its complex three-dimensional geometry.

Note that a recent comparison of epitaxial and heterogeneously integrated GaSb-based lasers revealed superior performances of epitaxial lasers in terms of threshold current density and maximum operating temperature^23^. We thus expect the same to be true for the lasers studied here that are grown on a PIC.

### Light coupling into the SiN waveguide

**Fig. 5:**
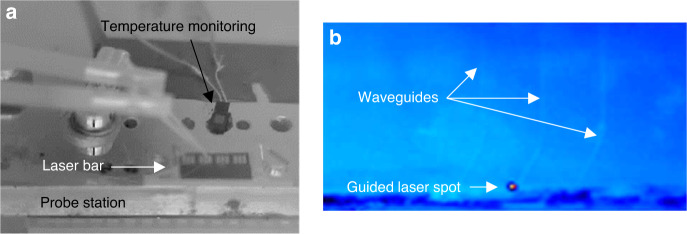
IR camera images of the integrated devices. **a** IR image of the laser bar on the probe station. **b** colored IR image of the waveguides, evidencing light exiting from a waveguide when the corresponding DL is operated at 1.5 × *I*_th_

The preceding section has shown that the quality of the epitaxial heterostructure grown on the Si PIC is comparable to that on plain Si wafers. The next step consisted in evaluating whether the laser light was coupled to the waveguides in this butt-coupling configuration. First, an IR camera was used to image the whole setup (Fig. 5a) and the waveguides (Fig. 5b). Great care was taken to avoid that stray or scattered light arising from the emission cone of the laser facet (typically ∼120° × 60°) hit the camera. Figure 5b shows that a light spot exits the waveguide when the corresponding DL is operated above the laser threshold. This unambiguously shows that (i) laser light is coupled into the waveguide and (ii) light does propagate through the waveguide.

**Fig. 6:**
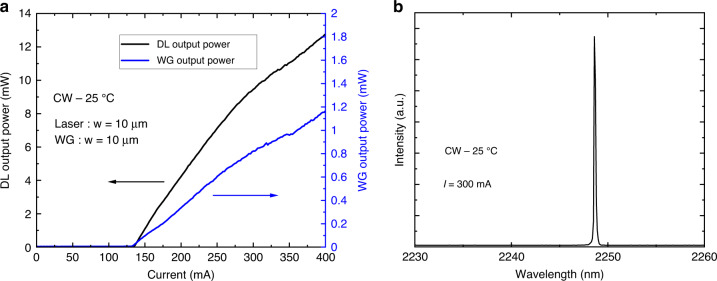
*L–I* curves and emission spectrum taken from a DL grown on a Si PIC. **a** The black curve represents the output power emitted by the back facet of the DL whereas the blue curve was measured at the exit of the waveguide facing the same DL. **b** Emission spectrum recorded from the DL at room temperature in the continuous wave regime at ∼2 × *I*_th_ drive current

During the second set of investigations, the waveguide output power as a function of the DL drive current was measured and compared to the optical power emitted through the cleaved backside facet of the DL. We display in Fig. 6a) the corresponding *L–I* curves measured in the CW regime at room temperature for a chip where both the DL ridge and waveguide are 10 µm wide. Both curves show a threshold current intensity at 135 mA, which confirms laser emission from this chip, and the threshold does not seem to be affected by any light being reflected from the dielectric stack toward the laser heterostructure. Figure 6b shows that the emitted wavelength is near 2.3 µm, as expected from the heterostructure design. In addition, while 12.7 mW output power are measured from the cleaved facet, around 1.2 mW are measured at the exit of the waveguide, which gives a minimum value of about 10% transmitted light in this butt-coupling configuration. The propagation losses in SiN being low in this wavelength range (0.1–2.5 dB/cm around 2 µm)^68,69^, we consider that most of the measured losses are insertion losses. We thus conclude that the upper value for the insertion losses in our system is 10 dB.

**Fig. 7:**
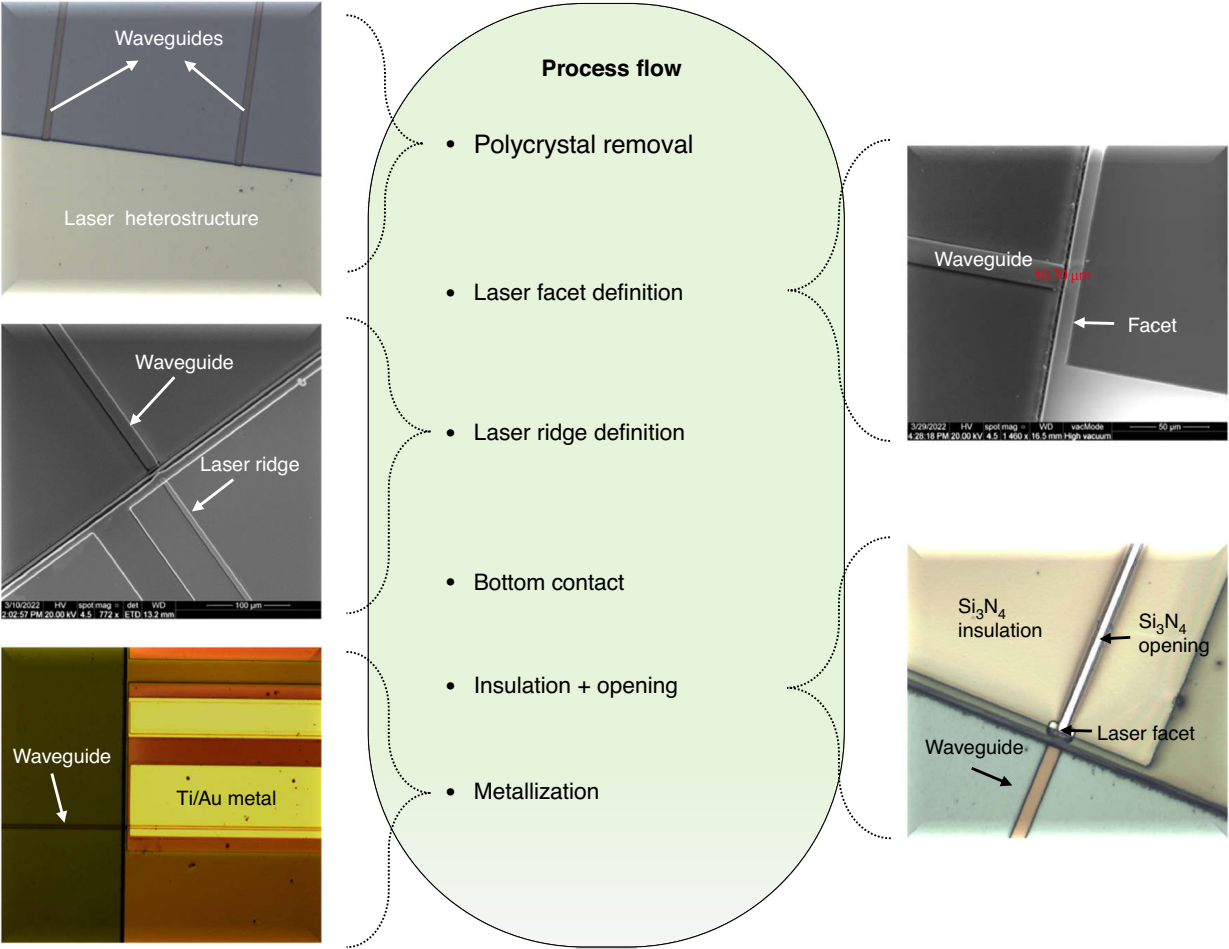
Fabrication of the diode lasers grown on the Si PIC. The pictures document the progression of the process flow in sequence from left to right, top to bottom: polycrystal removal, laser etched-facet definition, laser ridge definition, bottom contact definition, insulation deposition and contact opening, contact metal deposition

The measured coupling efficiency (∼10%) is in line with theoretical calculations analyzing the impact of an air gap between the laser and waveguide facets which concluded that the coupling efficiency rapidly drops with the gap width^53^. As mentioned above, removal of the parasitic polycrystal material and DL facet etching inevitably creates an air gap at the interface between the DL and the waveguide devices which, in this sample, is around 15 µm (Fig. 3a). This air gap has thus two origins, one is the polycrystalline material that deposits at the III–V/dielectric interface during the laser epitaxy, the other one is the definition of the cavity mirrors. The first origin is unavoidable and gives a lower limit for the gap roughly comparable to the laser thickness, i.e., ∼3 µm. The second origin can be drastically reduced by refining the laser processing steps, e.g., by using focused ion-beam lithography rather than photolithography and ICP-RIE to define the cavity mirrors^70^. Even though the simulated coupling efficiency does not improve much until the air gap is narrower than ∼2 µm, these simulations show that it drastically increases when the gap is filled with a high refractive index (*n*) material^53^. Filling the gap with amorphous silicon (*n* = 3.44) or chalcogenide materials (*n*∼2.5) would allow a coupling efficiency higher than 50%^53^, but filling a narrow gap with these materials might prove cumbersome. In contrast, filling the gap with polymers such as PMMA or BCB (*n*∼1.5), which is easy to do given their liquid state before polymerization, would already give a coupling efficiency as high as 40%^53^.

## Discussion

We have developed a complete process flow to obtain a semiconductor laser grown on a patterned Si photonics platform with light coupled into a waveguide. The different challenges (PIC fabrication and patterning, regrowth on a pattern PIC, etched-facet laser processing in recessed areas, etc.) due to the particular architecture of the final devices were all overcome. The test vehicle was a mid-IR GaSb-based diode laser directly grown on a pre-patterned Si photonics wafer equipped with SiN waveguides clad by SiO_2_. Both qualitative (IR images) and quantitative (light output power) measurements demonstrated that the laser light was butt-coupled into the waveguides, with a coupling efficiency in line with theoretical calculations. The high threading dislocation density still present in such DLs will surely impact their lifetime. However, various strategies proved efficient to decrease this density with various semiconductor materials^24,43^, and they will be implemented in the future with GaSb DLs. In addition, we have recently demonstrated that mid-IR interband cascade lasers^44^ and quantum cascade lasers^37,42,71^ are essentially immune to dislocations. This opens the way to long-lifetime mid-IR monolithic lasers grown on Si platforms for integrated sensing chips.

The approach presented in this article can be extended to any semiconductor materials system provided the antiphase domain problem can be solved, which is the case with most III–V systems nowadays^24^, and the various processing steps are established, which does not pose fundamental issues. In addition, it can be scaled up to any Si-wafer size up to at least 300 mm diameter, epitaxial reactors being available.

Altogether, this work thus solves a longstanding problem, and it lays the foundation for future low-cost, large-scale, fully integrated photonic chips.

## Materials and methods

### Si photonic wafer preparation

The wafers were processed in the 100 mm CMOS line at the Tyndall National Institute. 3.9 µm of oxide (SiO_2_) was grown on the silicon wafers using a thermal wet-oxidation process. A 800 nm thickness silicon nitride (SiN) layer of was then deposited using plasma-enhanced chemical vapor deposition (PECVD). The waveguides were patterned using stepper lithography, and then etched into the SiN layer using ICP plasma etch, with soft-landing onto the underlying SiO_2_ layer. A 1000 nm thickness cladding layer of oxide (SiO_2_) was then deposited by PECVD. The area for the epitaxy of the GaSb was then patterned by stepper lithography, and a two-stage etch process used to expose the Si surface; firstly an ICP plasma etch was used to remove the majority of the thickness of the dielectric stack (SiO_2_/SiN/SiO_2_), leaving ~200–300 nm of SiO_2_ remaining, before a wet etch using buffered oxide etch (BOE) removed the last of the SiO_2_.

The stress in the thick oxide layers created wafer bow which was compensated by depositing the same layer stack on the underside. The compensating layers were removed after dicing. The recess dimensions were 16 × 2.5 mm² and the S-bend offset was 300 µm.

### Diode laser design and epitaxial growth

The core of the laser heterostructure is typical for GaSb-based DLs emitting near 2.3 µm. The active zone was made of two Ga_0.67_In_0.33_As_0.08_Sb_0.88_ quantum wells (QWs) confined by Al_0.25_Ga_0.75_As_0.02_Sb_0.98_ barrier layers. This ensemble is embedded within an Al_0.25_Ga_0.75_As_0.02_Sb_0.98_ waveguide, itself clad by Al_0.9_Ga_0.1_As_0.07_Sb_0.93_ layers. All Al_x_Ga_1-x_As_y_Sb_1-y_ layers are lattice-matched, whereas the Ga_0.67_In_0.33_As_0.12_Sb_0.88_ QWs suffer a ∼1.5% mismatch, with respect to the GaSb lattice parameter. The laser structures were grown on the GaSb-on-Si PIC templates by solid-source MBE under conditions similar to those used for GaSb DLs grown on GaSb substrates. After GaSb deoxidation at ∼550 °C, a 500 nm GaSb buffer layer was grown at 500 °C. Then, the temperature was set at 470 °C for growing an *n*-type, 500 nm thick InAs_0.92_Sb_0.08_ layer, an additional 500 nm thick *n*-type GaSb buffer layer and the laser heterostructure. The InAs_0.92_Sb_0.08_ layer serves as both a marker during DL processing and as the back-contact layer to the DLs. No dislocation filtering layer was inserted in the buffer layers.

### Diode laser fabrication process

Ridge DLs were processed using standard photolithography and inductively coupled plasma reactive ion etching. Electrical insulation and protection of the etched sidewalls were obtained using SiN deposited by plasma-enhanced chemical vapor deposition. Both *p*- and *n*-contacts were taken on the epitaxial structure. The *p*-contact was taken on the top ridge while the n-contact was taken on the InAs_0.92_Sb_0.08_ layer located within the buffer layer stack. The *n*-contact is typically about 40 µm away from the ridge. This geometry avoids driving the current through the highly defective III–V/Si interface and ensures higher DLs performance^72^. Ti–Au and AuGeNi were used as contact metals for the *p*- and *n*-type contacts, respectively. The etched facets were fabricated as previously described^64^. No optical treatment was applied to the facets. The laser bars were then soldered substrate-side down on Cu-heat sinks for being tested on a probe station.

The process flow is shown in Fig. 7.

## Data Availability

The data underlying the results presented in this paper are not publicly available at this time but may be obtained from the authors upon reasonable request.
